# Reflection Supports Newly Graduated Nurses' Professional Development When Transitioning Into Practice

**DOI:** 10.1111/jocn.17772

**Published:** 2025-04-10

**Authors:** Magdalena Lindblom, Jessica Höglander, Anna Letterstål, Marie‐Louise Södersved Källestedt, Margareta Asp, Margareta Widarsson

**Affiliations:** ^1^ School of Health, Care and Social Welfare Mälardalen University Sweden; ^2^ Centre for Innovation, Research and Education Region of Västmanland Sweden

**Keywords:** caring science, focus group, intervention, introduction programme, newly graduated nurses, professional development, reflection, support, transition

## Abstract

**Aim:**

To describe newly graduated nurses' experiences of reflection as a support for professional development during the initial months of their transition while caring for patients in a hospital setting.

**Design:**

A qualitative descriptive design.

**Methods:**

Four focus groups with 20 newly graduated nurses participating in a professional development programme at aregion in Sweden were conducted in 2023. The data were analysed using qualitative content analysis.

**Findings:**

The analysis identified one main category: Reflection supports newly graduated nurses' professional development during their transition. This main category includes three generic categories: (1) Reflection with peers in a regularly structured dialogue group strengthens the professional role; (2) reflection with experienced healthcare instructors in learning activities enhances the mastery of care tasks; and (3) reflection with experienced colleagues in the workplace enhances task performance. Structured reflection in dialogue groups and interactive learning activities within the Professional Development Programme facilitated deeper reflections on caring experiences.

**Conclusions:**

Newly graduated nurses reported that regularly structured reflection, adequate space, and established trust were essential to their professional development. While the professional development programme provided opportunities, variations in the workplace environment led to unequal conditions for reflective practice.

**Implications for the Profession:**

Addressing the need for reflection among newly graduated nurses is crucial for organisations to facilitate their transition. Establishing structures for reflection on caring experiences within introduction programmes can support their professional development.

**Impact:**

Reflective practice in complex and challenging hospital settings can support the professional development of newly graduated nurses.

**Reporting Method:**

The Consolidated Criteria for Reporting Qualitative Research (COREQ) was adhered to.

**Patient or Public Contributions:**

No patient or public contributions.

**Summary**

- What does this paper contribute to the wider global clinical community?
  ○ Reflection supports the professional growth of newly graduated nurses (NGNs) and should be integrated into both introduction programmes and workplace environments.
  ○ Peer dialogue groups are essential for reflection, providing emotional support and reinforcing NGNs' professional identity.
  ○ Managers play a crucial role in facilitating reflective opportunities in the workplace by ensuring the necessary prerequisites for NGNs to engage in meaningful reflection.

## 1 Introduction

The transition from student to registered nurse (RN) can be overwhelming for newly graduated nurses (NGNs), as they must navigate the complexities of being responsible for patient care. Previous research indicates that the fast‐paced work environment, coupled with a heavy workload and inadequate staffing, presents significant challenges (Reebals et al. 2022; See et al. 2023). This period of transition can be experienced as a shock for NGNs during their professional role transition (Duchscher 2008). These challenges in the health care environment underscore the importance of providing sufficient support to NGNs to ensure a successful transition. During the transition period, NGNs need a supportive environment where they can discern which aspects of the theoretical knowledge gained during their nursing education are applicable in practice (Bisholt 2012). The integration of theoretical knowledge into practice is a key learning strategy (Eklund et al. 2021; Rush et al. 2019). Therefore, it is essential to merge theoretical and practical knowledge to facilitate professional development.

Reflection is emphasised as a means to integrate theory with practice and support NGNs in their new professional roles. Previous research indicates that reflection on caring experiences in patient care plays a vital role in supporting professional development (Bindon 2017; Ekebergh et al. 2018). To provide high‐quality care, NGNs must be supported to ensure they have the necessary knowledge and competence to carry out their responsibilities (Bindon 2017; Page et al. 2020). Ensuring patient safety also requires that NGNs receive adequate support from their colleagues. Without this support, many may choose to leave their workplace or even the profession entirely (Kaldal et al. 2023). This issue is widespread and contributes to the ongoing nursing shortage, which not only jeopardises patient safety but also threatens the future availability of healthcare resources (WHO 2022). Providing support for professional development during the NGNs´ transition has been demonstrated to reduce attrition rates (Reebals et al. 2022). This highlights the importance of having adequate prerequisites in place to support NGNs, as a recognised lack of guidance can lead to low job satisfaction. By offering appropriate support, a sustainable transition can be achieved, encouraging NGNs to remain in the profession.

## 2 Background

In Sweden, nursing students must complete a bachelor's degree in nursing to qualify as RNs. For NGNs, entering the workforce brings considerable pressure and high expectations from both colleagues and patients to manage the responsibilities of an RN. Previous research indicates that NGNs often question their knowledge and skills (Kaldal et al. 2023; Sterner et al. 2023), and express concerns about making mistakes that could potentially harm patients (Willman et al. 2021). This fear is especially pronounced during the first three months (Gustavsson et al. 2020). NGNs experience elevated stress levels in hospital settings, where they face high‐intensity and complex care encounters. Prolonged or particularly intense stressors can lead to stress‐related ill health issues (Reebals et al. 2022; See et al. 2023). Therefore, support structures are essential to help NGNs navigate their transition into practice, in order to manage stress and ensure patient safety.

Support from experienced RNs in the workplace is crucial for helping NGNs manage responsibilities, overcome challenges, and foster their professional development (See et al. 2023; Widarsson et al. 2020). However, a shortage of experienced RNs to provide guidance can make this transition particularly challenging (Willman et al. 2021). Early in their careers, NGNs report feelings of loneliness and a sense of not belonging to the profession (Baharum et al. 2023; Wakefield et al. 2023). It is therefore imperative that NGNs are provided with support in order to feel included in the profession. Despite the challenges of transition, previous research shows that NGNs remain optimistic and motivated to develop their skills to provide optimal patient care (Baharum et al. 2023; Widarsson et al. 2020). They demonstrate a strong willingness to grow (Berglund et al. 2022), but face difficulties in developing the professional competence necessary to meet the daily demands of an RN (Widarsson et al. 2020).

To support the professional development of NGNs, it is essential to provide the necessary guidance for them to navigate their role as novice learners, enabling progression that encompasses all aspects of patient care (Baharum et al. 2023; Eklund et al. 2021). The journey from novice to expert involves acquiring both knowledge and practical experience, which fosters the development of the nurse's professional role (Benner 2004). This highlights that professional development takes time since experience is needed. Reflection on clinical experiences and theoretical knowledge is a way to further one's knowledge and increase professional development to meet the demands of an RN in clinical practice (Pool et al. 2016). To help NGNs meet these demands, introduction programmes designed to support their early careers in hospitals are commonly offered by healthcare organisations (Reebals et al. 2022). These programmes aim to stimulate learning, foster development, and integrate NGNs into the profession (Berglund et al. 2022; Rush et al. 2019) as well as provide a foundation for regular support for NGNs, which is essential for a successful role transition.

Another critical factor in facilitating professional development is teamwork and engaged leadership (Page et al. 2020). NGNs require support from colleagues in their daily work, including proper introductions and supervision, to meet care expectations (See et al. 2023; Wakefield et al. 2023). However, such support in workplaces is not always well coordinated (Eklund et al. 2021) and the lack of appropriate guidance can hinder the development of new skills (Reebals et al. 2022). To ensure NGNs' professional development, it is crucial they are provided with opportunities to engage with their professional role by actively analysing their actions in practice and applying theoretical knowledge (Sterner et al. 2023), which can be achieved through reflection (Rush et al. 2019; See et al. 2023). Reflection represents a valuable learning opportunity that fosters awareness of one's competence and actions, offering potential for improvement (Bengtsson 1998). Engaging in reflection with colleagues, particularly concerning complex situations, is essential for professional development (Bindon 2017; Ekebergh et al. 2018). Peer reflection on transition experiences is recognised as a valuable source of support (Rush et al. 2019; See et al. 2023).

Reflection is a thoughtful process that verbalises and concretises cognitive distance from caregiving actions, allowing one to shift between different perspectives (Bengtsson 1998). It can help identify gaps in learning needs and facilitate professional development throughout a nurse's career (Bindon 2017). One strategy to support reflection is Kolb's Experiential Learning Theory, which emphasises learning from holistic care experiences for professional development (Kolb 1984). Opportunities for in‐depth reflection and knowledge sharing are essential; however, these opportunities are often insufficient in the fast‐paced hospital environment (Widarsson et al. 2020). This is concerning, as reflection can help NGNs express thoughts and emotions associated with emotional distress, transforming experiences into valuable insights that enhance their understanding of patient care and increase self‐awareness (Bolden et al. 2011). Including ethical and emotional dimensions in reflection can enhance NGNs' professional development of softer skills needed in patient care rather than focusing solely on technical tasks. Despite the importance of reflection, there is limited research on its role in supporting NGNs' professional development. Therefore, this study aimed to describe NGNs' experiences of reflection as a support for professional development during the initial months of their transition while caring for patients in a hospital setting.

## 3 Method

### 3.1 Design

A qualitative descriptive design was chosen to gain a deeper understanding of the participants´ experiences (Polit and Beck 2020). Focus groups (Morgan 1997) were used for data collection, and an inductive content analysis was conducted (Elo and Kyngäs 2008). To achieve credibility, the study followed the COREQ 32‐item checklist (Tong et al. 2007).

### 3.2 Study Setting and Recruitment

A Professional Development Programme (ProDeveloP) was implemented in 2023 in a hospital setting within a region in mid‐Sweden. The ProDeveloP included eight educational days and six dialogue group sessions, utilising a pedagogical strategy aimed at promoting reflection and facilitating learning. All 62 eligible NGNs who participated in the ProDeveloP in 2023 were invited to participate in the focus groups during one of the educational days by the first author. A total of 29 NGNs volunteered to participate. The focus groups were scheduled at a time convenient for most participants. However, nine declined due to high workload or scheduling difficulties. Ultimately, 20 NGNs participated in four focus groups, each comprising four to seven participants (Table 1). The NGNs comprised two males and 18 females, aged between 27 and 54 years, with four to nine months of professional experience as RNs.

**TABLE 1:** Overview of the participants who took part in the focus groups.

Focus group (*n* = 4)	1 (Cohort 1)	2 (Cohort 1)	3 (Cohort 2)	4 (Cohort 2)
Participants (*n* = 20)	NGN 1–7 (*n* = 7)	NGN 8–11 (*n* = 4)	NGN 12–16 (*n* = 5)	NGN 17–20 (*n* = 4)

### 3.3 Data Collection

Qualitative data were collected from the NGNs who participated in focus groups. A moderator (first author) and an assistant moderator (last author) conducted four face‐to‐face focus groups. The assistant moderator took notes during the sessions to ensure accurate attribution of the participants' comments. Data were collected through participant interaction to explore the groups' perceptions from different perspectives and to identify contrasts in their experiences (Morgan 1997). The participants were encouraged to freely share their diverse thoughts, with the moderator only intervening when necessary. A semi‐structured interview guide was developed and employed, comprising an introduction, along with key, concluding, and follow‐up questions (Table 2). Prompts were used when appropriate to elicit further clarification or explanation of the objective. The focus groups were held in a reserved room at a hospital in May and December 2023 and lasted 105–115 min. All interviews were recorded and transcribed verbatim, with the participants anonymised using code identifiers.

**TABLE 2:** Semi‐structured interview guide.

Introduction question	What are your descriptions or thoughts on the concept of professional development?
Key questions	What are your experiences of participating in the programme? What has been valuable content in the programme for your professional development? What valuable support have you received in your worksite as a nurse for your professional development? What experiences do you have of opportunities for reflection? What experiences do you have from the dialogue group? Is there support that you need that you have not received? Is there a suggestion of support that can be developed?
Ending questions	Can you describe the most important thing we discussed today?
Follow‐up questions, probes	Can you explain more? Give an example of a situation. Can you develop your description? More specific or detailed?

### 3.4 Data Analysis

The focus groups were analysed systematically using inductive qualitative content analysis, following the three main phases of preparation, organisation, and reporting (Elo and Kyngäs 2008). In the initial stage, the first author and the last author read the transcribed data several times to gain an overall understanding. Units of analysis relevant to the aim were identified and incorporated into a coding sheet. Open coding was conducted to organise the data. Headings and notes were written in the margins. The codes were then freely grouped manually as part of the initial abstraction process. To reduce the number of categories, groups based on similarities were organised into subcategories. These subcategories were named according to their content characteristics and subsequently organised into higher‐order generic categories and finally into a main category, providing a comprehensive understanding of the NGNs' experiences. Table 3 provides an example of the analysis process. This iterative process facilitated both the understanding and description of the underlying meaning of reflection as a support for professional development.

**TABLE 3:** An example of a subcategory, showing the abstraction process of organising data into categories.

Units of analysis	Open coding	Subcategory
I think it worked well to raise a problem where you dig down a little bit and hear that others have the same experience, but also hear their different thoughts about the experience, and twist and turn it. Can you see it that way? Could we do that? How would it feel if we did this? What will you take with you next time? This was extremely rewarding and useful. *–Participant NGN3*	When raising and digging deeper into problems during dialogue groups, hearing others' input was supportive	Processing experiences together in the group facilitates mutual support
I raised my problem and got to hear everyone's opinions in the group of new nurses how they would have managed it, and there were good ideas. It has helped me to process problems that I have in the workplace to find a solution. *–Participant NGN12*	In the dialogue group, they could raise problems and process them with support from others, hear their opinions, and give innovative ideas for solutions	
For example, if I am faced with something completely new, What should I do? For example, about the role clarity, How should I think here? Like if you are unsure. I can be confident in myself but at the same time, I don't know how to act. Is it really my nurse's responsibility? There are some questions like that. All the participants in my group gave some advice and tips, that maybe you can do this next time? This helped me get a clearer picture of my role. *–Participant NGN11*	They got support from others in the dialogue group when reflecting on how to handle the new situation of being a nurse in case of uncertainty, which increased their role clarity	

### 3.5 Ethical Considerations

The study was approved by the Swedish Ethical Review Authority (Dnr. 2022–05682‐0). Before their participation, all participants were provided with both verbal and written information about the study by the first author, including that their involvement was voluntary, that they could withdraw at any time, and that confidentiality would be maintained. All participants were informed about the ethical considerations and gave written informed consent before participating. No prior relationships existed between the participants and the researchers before the study. While the participants in the focus groups knew each other from ProDeveloP and therefore were not anonymous to each other, the content from the focus groups was treated confidentially. All participants were anonymised in the collected data and the reporting of the findings. A data management plan was finalised in the spring of 2023 to ensure secure data storage and compliance with the EU's General Data Protection Regulation.

### 3.6 Rigour and Reflexivity

The co‐authors engaged in self‐reflection and reflexive methodology discussions throughout the research process (Alvesson & Sköldberg, 2018). When planning the study, the co‐authors reflected on the knowledge gap and challenges of collecting empirical material, recognising its great value to the study's aim. Before the focus groups, the researchers engaged in reflexive discussions about their extensive experiences in practice and teaching at the School of Health, Care, and Social Welfare. This enabled them to be mindful of their pre‐understandings and facilitated open and honest discussions. At the end of the focus groups, a designated period was allocated for summarising the content. During this time, participants were allowed to verify the accuracy and to add or clarify experiences, thereby ensuring affirmation. After the first focus group, a discussion between the moderators regarding the content and saturation was held, and by the fourth focus group, no new information emerged.

The trustworthiness of qualitative content analysis (Elo et al. 2014) was considered throughout the analysis process. The co‐authors engaged in reflexive discussions on multiple occasions to reach a consensus regarding the categorisation. During the writing‐up of the findings, interpretive steps were employed to abstract the content, ensuring that no meaning was lost or overlooked. To maintain the accuracy and transparency of the categorisation and findings, the researchers frequently returned to the units of analysis. The inclusion of quotations in the findings reinforces both the transparency and credibility of the content.

## 4 Findings

The findings of this study are based on the NGNs' experiences of reflection during their transition from student to professional RN in a hospital setting in mid‐Sweden. The main category—Reflection supports NGN' professional development during their transition—highlights that reflection plays a crucial role in supporting NGNs' professional development throughout this transition. Three generic categories further describe the different contexts and individuals involved in reflection that facilitate this development: Dialogue groups with peers, Educational sessions with healthcare instructors, and Workplace interactions with experienced colleagues. Self‐reflection was also practised across all these contexts. These generic categories are drawn from nine subcategories, which provide detailed insights into how reflection was implemented and experienced (Table 4).

**TABLE 4:** Overview of one main category, three generic categories, and nine subcategories.

Main category	Generic category	Subcategory
Reflection supports newly graduated nurses' professional development during their transition	Reflection with peers in a regularly structured dialogue group strengthens the professional role	Recognising each other's situation builds trust in the group Processing experiences together in the group facilitates mutual support Listening actively to others in the group broadens one's perspectives
	Reflection with experienced healthcare instructors in learning activities enhances the mastery of care tasks	Engaging in interactive learning activities facilitates comprehension of diverse care topics Participating in skills training in a simulated setting increases the readiness to manage professional work Acquiring knowledge from evidence generates new insights for clinical practice
	Reflection with experienced colleagues in the workplace enhances task performance	Working in an open collaborative climate includes paradoxes in managing work Relying on experienced registered nurses when uncertain requires certain prerequisites Experiencing professional work in clinical practice facilitates professional growth over time

### 4.1 Reflection With Peers in a Regularly Structured Dialogue Group Strengthens the Professional Role

Reflection within the dialogue group offered the NGNs a structured opportunity to reflect on their caregiving experiences in the hospital context and learn from them. Engaging with peers who were in similar situations and understood the challenges of being new to the profession provided invaluable support and emotional reassurance.

#### 4.1.1 Recognising Each Other's Situation Builds Trust in the Group

This mutual understanding fostered trust among group members, enabling the NGNs to support one another as they reflected on their workplace experiences. It was comforting to realise that others faced similar challenges, confirming that feelings of insecurity were a common part of transitioning into the profession. This sense of belonging and emotional support allowed the NGNs to engage in honest, vulnerable reflection at a depth that they found difficult to achieve in the workplace.

In this group, we had the exchange of being able to ventilate or listen to each other's thoughts and experiences, which I think has contributed to a safe forum for many, as I am the only new graduated nurse in my workplace, and I have no colleague to reflect with in the same way there. (NGN 7)

The structure of reflection within the dialogue group was described as needing modifications during the first sessions to help the NGNs become better acquainted with each other and to foster engagement. The participants expressed a preference for allowing all the NGNs to share their experiences from the beginning, rather than focusing solely on a few individuals. Furthermore, it was described as advantageous for the two experienced RNs, who acted as dialogue facilitators, to highlight key areas for reflection and share more of their own experiences as RNs. Despite the need for these potential improvements, the dialogue facilitators effectively supported peer reflection, helping the NGNs derive professional meaning from their experiences and instilling hope for their future growth into experienced practitioners.

#### 4.1.2 Processing Experiences Together in the Group Facilitates Mutual Support

Reflections within a trusting environment in the dialogue group enabled the NGNs to process their thoughts with peers who understood their circumstances. Experiences related to caring for patients with complex needs were discussed with a certain emotional distance, allowing for more objective reflection. Verbalising these experiences helped make them more concrete, establishing a foundation for deeper analysis.

I think it worked well to raise a problem where you dig down a little bit and hear that others have the same experience, but also hear their different thoughts about the experience, and twist and turn it. Can you see it that way? Could we do that? How would it feel if we did this? What will you take with you next time? This was very rewarding and useful. (NGN 3)

In processing experiences, the NGNs gained valuable insights into what had proved effective and beneficial for others. They recognised that they had collectively identified solutions within the group, enhancing their ability to navigate and overcome professional challenges associated with understanding complex patient care and their limitations regarding professional responsibilities. Through structured reflection, the NGNs developed a more positive outlook as they realised they had successfully managed their work. This positive reinforcement and validation provided emotional support and increased their confidence in their achievements. “I have experienced this, it's my experience, but the others can help me maybe not be so upset, sad or disappointed but proud too for that matter, I managed this” (NGN 13). As they processed their experiences during reflection, they gained new insights and a deeper understanding of their responsibility as RNs in clinical settings, which strengthened their professional roles. “Just hearing how we all reflected in the groups has, yes, helped me in my role clarity, because it was something we have talked a lot about, I have been able to use that in my work” (NGN 6). This reflective process supported their professional development and reinforced their willingness to share knowledge within the workplace, facilitating workplace advancements. They felt empowered to question and address difficulties observed in patient care, including setting goals to improve patient safety and the quality of care while navigating the demanding circumstances of a stressful work environment.

#### 4.1.3 Listening Actively to Others in the Group Broadens One's Perspectives

When unique experiences were shared during reflections in the dialogue group, the NGNs were exposed to different contexts and additional perspectives, which helped them discover alternative strategies for addressing challenges in their professional roles. This exchange stimulated self‐reflection and enhanced their capacity to manage future responsibilities. “I am quite bad at, or I do not think about reflecting. Hmm now I did this, next time I will probably do that. I did not think like that, but the group sessions have made me think more” (NGN 8). As the NGNs developed the ability to listen and reflect more deeply, they became less prone to prematurely identifying solutions, thereby enhancing their self‐awareness. The process of self‐reflection not only fostered personal growth but also contributed to their professional development. “The dialogue group has given me a lot, actually just to develop in my profession in many ways” (NGN 4). Overall, the dialogue group served as a valuable source of regularly structured, in‐depth reflection, significantly supporting individual professional development during their transition into practice.

### 4.2 Reflection With Experienced Healthcare Instructors in Learning Activities Enhances the Mastery of Care Tasks

Opportunities for reflection within learning activities during ProDeveloP educational days were crucial. These opportunities enabled the NGNs to understand better how the expertise and experience shared by healthcare instructors could be applied in their specific context, thereby enhancing their mastery of daily care tasks.

#### 4.2.1 Engaging in Interactive Learning Activities Facilitates Comprehension of Diverse Care Topics

Through exchanges during the educational days of ProDeveloP, the NGNs were engaged in reflective practice on the expert knowledge and experiences drawn from various clinical settings. This process was essential for translating the broad theoretical knowledge presented during the learning activities into a deeper comprehension of its practical relevance and application to their specific work context.

I find that these times when we had group discussions, I learn better that way when it becomes more practical than sitting and listening. I found it rewarding when we also had general discussions as well, that you exchange views with each other. (NGN 2)

Healthcare instructors who incorporated opportunities for reflection were more highly regarded than those who relied predominantly on passive learning through lectures. The extent to which reflection was integrated varied between different sessions, and the NGNs stated that reflective practice could have been given greater emphasis. They voiced a preference for healthcare instructors to include more reflective discussions from the experienced RN perspective, as well as drawing upon their own experiences of clinical expertise.

During the education days, we had a lecture. We had read a lot about it during the nursing education. Then maybe it would have been better to get a few more examples of what I do as a nurse or about patients' symptoms rather than what the subject means. I have heard that before, so I was expecting it to be more about what we do in practice rather than in theory. (NGN 19)

The knowledge gained from these learning activities was invaluable for mastering care tasks, thus contributing significantly to their professional development. Furthermore, these learning activities encouraged the NGNs to continue reflecting on a wide range of topics with their colleagues in the workplace.

#### 4.2.2 Participating in Skills Training in a Simulated Setting Increases the Readiness to Manage Professional Work

The NGNs were provided with invaluable opportunities for reflection alongside experienced healthcare instructors on various care tasks and acute scenarios. This reflective practice enhanced their ability to apply available evidence in real work situations, improved their communication skills within teams, and strengthened their leadership role as RNs. It is important to note that the NGNs experienced limited opportunities for skills training during clinical placements or nursing school, with even fewer during the pandemic. Reflection during skills training facilitated a deeper understanding of nursing care tasks, alleviated feelings of difficulty, and helped them manage time constraints more effectively. Additionally, it increased their ability to respond more professionally in acute situations.

On the day we practised A‐E of low blood pressure and high pulse, it became easier to understand when you went through and talked about it. Then you felt more professional, when you said as a team leader ‘lift the patient's legs and connect a drip’. So you need to be reminded. (NGN 8)

Reflection during the skills training increased their confidence in handling professional care tasks and acute situations, ultimately improving the quality of care using methods grounded in evidence.

#### 4.2.3 Acquiring Knowledge From Evidence Generates New Insights for Clinical Practice

The knowledge gained from evidence presented during the ProDeveloP education days, when applied within a clinical context, was vital for the NGNs in enhancing their mastery of care tasks. Through self‐reflection, the NGNs were able to clarify the knowledge‐based content presented, integrating it with their own understanding and experiences. They reflected on the potential applicability of this new knowledge in managing various care situations in their professional practice. Self‐reflection was seen as improving the quality of patient care.

You have gained more knowledge, and you have been able to absorb knowledge that you can then apply yourself in your work. And be able to reflect when health instructors give a lecture because you can picture a situation with a previous patient and apply the knowledge gained to improve how you handle similar situations with future patients. (NGN 10)

Through self‐reflection on the content from their education days, the NGNs gained greater confidence and a deeper understanding of how to master care tasks. This, in turn, led to significant professional growth, benefiting both the NGNs and the workplace. They expressed their appreciation of participating in ProDeveloP and highlighted the need for structured reflection. Additionally, they voiced a desire for the programme to be extended to more experienced RNs.

### 4.3 Reflection With Experienced Colleagues in the Workplace Enhances Task Performance

When faced with uncertainty in performing care tasks, reflecting with experienced colleagues was essential for managing the demands of caring for patients with complex needs in a hospital setting. However, such reflection often centred predominantly on completing care tasks, rather than on optimising patient care or addressing ethical considerations. In the workplace, the necessary conditions for structured reflection, such as trusting relationships, were lacking. This hindered deeper reflection on caregiving and limited the opportunity to share knowledge for professional growth. This misalignment with the NGNs' expressed need for reflection on compassionate care and ongoing professional development was a noted challenge.

#### 4.3.1 Working in an Open Collaborative Climate Includes Paradoxes in Managing Work

While it was acknowledged in the workplace that the NGNs needed time to learn, they were often expected to manage the same patient load and complexity as more experienced RNs after only a few weeks. This expectation did not match their capacity and left little room for reflection and professional growth. Moreover, there was often a lack of managerial emphasis on reflection and professional development, despite the NGNs expressing a clear need for reflective practices as newcomers. Although policies suggested a follow‐up meeting with managers to reflect on their transition and professional development, such meetings rarely occurred within the first months. The NGNs reported that reflecting on their experiences after six months or more felt too late to be meaningful. Despite challenges with the prerequisites for structured reflection, it was accepted within the workplace to ask for help, facilitating spontaneous daily reflection with colleagues from various professions. However, these reflections tended to focus on care task performance rather than deeper professional or ethical issues. The availability of structured reflection varied, though reflection was always coordinated in response to serious incidents. During dedicated reflection meetings, the NGNs made efforts to adopt a proactive approach to vent work‐related thoughts and experiences.

In the reflection at work, you got support for these things that you have been thinking about. For me, it was so rewarding and good. I have tried to use it more. I talk more in the reflection now because I notice that it helps me. After all, it rounds off the day so well, that now I have said what I might have found difficult, then I can let it go when I go home. (NGN 15)

Despite being welcomed and socially included in the workplace, the NGNs reported challenges in trusting colleagues enough to share sensitive thoughts during reflection. These sessions often felt superficial, with the NGNs not always being honest about their experiences. “When reflecting with colleagues in a group in the workplace everyone says it was a good day even though you have not had a good day, it feels very superficial and not so transparent” (NGN 8). A lack of structure and frequent disruptions during reflection sessions negatively impacted the quality of the discussions. NGNs felt that using structured reflection questions could help elicit more genuine thoughts and emotions. Various outcomes were described when structured questions were used.

We sometimes have reflection that can be both unstructured, but sometimes it becomes more structured, but it is usually not that deep. But if our manager was there, more searching questions were asked like ‘What has been good about the day or what has been less good?’ Not just asking questions like ‘How has the day been? And you answer good. (NGN 11)

Reflection on the ethical dimensions of care was frequently missing from workplace discussions, which did not meet the NGNs' needs to process the emotional challenges they faced while caring for patients. However, they received support through reflection with experienced colleagues, which helped them gradually develop their ability to improve task performance.

#### 4.3.2 Relying on Experienced Registered Nurses When Uncertain Requires Certain Prerequisites

Finding time for reflection, or locating an experienced RN to reflect with, posed significant challenges for the NGNs. Spontaneous reflection with experienced RNs was crucial; a prerequisite for planning, prioritising, and performing tasks, particularly given the NGNs' limited professional experience. Reflecting during work shifts helped validate clinical judgements, supporting decision‐making until the NGNs developed greater confidence in their own abilities. Reflection was key for clarifying RN responsibilities, enhancing patient safety, and alleviating anxiety about making mistakes.

But if you are a bit unsure about something, did I do the right thing here really? Or did I think right? Or did it do wrong? Then it is nice to be able to talk to somebody who can give their view. A more experienced nurse has perhaps been through the same thing before as I am going through now. (NGN 12)

Opportunities for reflection with experienced RNs were inconsistent and varied across different workplaces. However, the NGNs found considerable room for reflection when they were assigned an RN as a clinical supervisor during their introduction period, which lasted between four and twelve weeks. They were encouraged to seek continued support after this introduction, and the more experienced RNs were generally willing to offer help. However, once the introduction period ended, it became challenging to find time and prioritise reflection in the fast‐paced work environment, particularly given the longer time the NGNs required to complete tasks. Moreover, due to the nursing shortage, it was often difficult to find an experienced RN for reflection. While temporary RNs occasionally provided pedagogical support and shared insights from their diverse experiences, their understanding of the specific workplace context was often limited.

The NGNs expressed a need for dedicated time and space to reflect on their individual development, even after several months in the role. Reflection with experienced RNs frequently lacked individual feedback on how they were managing the profession's expectations and leadership responsibilities. Having an experienced RN as a mentor over a longer period was described as highly valuable, providing emotional support and fostering professional development. “I had an experienced nurse as a mentor that I could talk to and bounce ideas off. It has helped me and I feel a bit better after the conversation” (NGN 17). However, the availability of mentors varied across workplaces, and scheduled meetings were often postponed due to heavy workloads. Having an experienced RN as a facilitator for dialogue groups within ProDeveloP created conditions for more structured, in‐depth reflection within the workplace. Facilitators were also able to highlight the NGNs' need for support within the workplace, a need often brought up during the dialogue groups.

#### 4.3.3 Experiencing Professional Work in Clinical Practice Facilitates Professional Growth Over Time

Initially, the NGNs relied on the experiences of their colleagues when reflecting on their own practice, as they lacked substantial personal experience in the specific clinical context. Self‐reflection on professional experiences within the workplace was crucial for helping the NGNs navigate the challenging transition at the start of their careers. It allowed them to process unresolved thoughts before heading home and to manage emotions that could otherwise disturb their sleep.

I usually try to self‐reflect on what was good and take it with me, and what was bad, I try to think, what could I have done differently? And then when I lock my car, I usually try to just shake it all off and not think about work until it is time again, so I can get on with it. So I have been doing that. In the car, I think about what I did and then it is fine. (NGN 16)

Although their foundational knowledge was primarily theoretical, self‐reflection on practical experiences was essential for improving performance and supporting individual professional development. By engaging in self‐reflection, the NGNs were able to identify their strengths and pinpoint areas for improvement, considering what they had done well and what could have been handled differently. Over time, this reflective process helped them better understand how to fulfil their role, manage various tasks, and respond to diverse care situations.

But everything is so new. I still think about the time, the first six months when you go to work and realise for yourself what your professional role is and what your tasks are. It somehow becomes clearer without perhaps getting support from anyone outside, just by working practically. So you learn for yourself what your role is just by thinking about it. (NGN 20)

Through self‐reflection on their professional experiences, the NGNs became aware of their development, enhancing their self‐awareness and fostering a sense of self‐belief. They experienced increased confidence and job satisfaction. However, they acknowledged that there was still much to learn and that their professional development would continue to evolve over time within the specific context of the workplace. Additionally, the NGNs highlighted the importance of self‐reflection for their professional development, while also recognising that reflecting with others in a trusting environment was equally vital for supporting their development.

## 5 Discussion

This study aimed to describe the NGNs' experiences of reflection as a support for professional development during the initial months of their transition while caring for patients in a hospital setting. The main findings emphasise that opportunities for self‐reflection and reflection with others were crucial for supporting the NGNs' professional development during this period. Professional development in this context refers to the ability to acquire and apply the competencies needed to fulfil the nurse role in clinical practice (Pool et al. 2016). This is further reinforced by the study's findings, which suggest that professional development is essential for managing the role's demands and navigating complex care tasks in specific work environments. In this study, the NGNs engaged in self‐reflection to translate theoretical knowledge into practical skills that meet the demands of clinical practice. Self‐reflection helped them clarify their thoughts and experiences, while also enhancing their ability to appreciate the different perspectives of others. These findings align with the assumption that self‐reflection facilitates a more comprehensive understanding of their work and enhances self‐awareness, fostering professional development (Bengtsson 1998). In addition to self‐reflection, both ProDeveloP and workplace‐based reflection with others played a key role in supporting the NGNs' professional development.

Reflections with peers, particularly within the ProDeveloP dialogue group, were described as especially valuable for strengthening their professional identity and supporting their development. This aligns with the findings of Bindon (2017) and Ekebergh et al. (2018), which show that reflecting on patient care experiences in dialogue with peers enhances professional growth. These structured reflection opportunities allowed the NGNs to process their experiences and apply insights from patient care. This finding aligns with Bengtsson's (1998) description of reflection as a thoughtful process of revisiting and concretising experiences. The study also indicated that structured reflection helped NGNs gain a deeper understanding of their nursing role, which is supported by previous research showing that introduction programmes offering regular process‐oriented reflection can enhance role clarity (Eklund et al. 2021). The dialogue group, facilitated by experienced RNs, provided the NGNs with a trusting and supportive environment. This sense of trust and belonging is critical for learning (Bisholt 2012), especially given that NGNs often report feelings of isolation (Baharum et al. 2023). Being part of a safe environment fosters professional development and helps NGNs provide competent and safe care (Kaldal et al. 2023). Furthermore, NGNs, with the benefit of reflection, can transform challenging experiences into valuable learning opportunities. Reflection has been shown to promote self‐awareness and transform emotionally charged experiences into valuable insights (Bolden et al. 2011). Emotional support, provided through peer reflection in the dialogue group, played a key role in helping the NGNs cope with the emotional challenges associated with caregiving. Such support is vital for professional development (Reebals et al. 2022).

The reflection facilitated by healthcare instructors during learning activities on ProDeveloP education day supported professional development, particularly in the mastery of care tasks. NGNs found skills training especially valuable, as many had received limited practical experience in certain areas. Previous research indicates that NGNs are eager to learn, and opportunities for reflection within learning activities are essential for their professional development (Widarsson et al. 2020). Such learning activities, embedded in introduction programmes, play a crucial role in fostering NGNs' professional development (Rudman et al. 2024; Willman et al. 2021). This study's findings align with these perspectives, highlighting NGNs' need for reflective opportunities in structured learning settings. To optimise professional development, learning activities must be tailored to address the specific needs of NGNs (Pool et al. 2016). The participants in this study expressed a need for reflection on how to apply general theoretical knowledge to specific clinical contexts to improve patient care. The integration of theoretical and practical knowledge has previously been identified as a crucial factor in the professional development of NGNs (Eklund et al. 2021; Rush et al. 2019). Supporting novice nurses, or advanced beginners, in this integration process is crucial to their growth and confidence in clinical practice (Benner 2004).

Moreover, the NGNs in this study expressed a desire for the continuation of education days and dialogue groups, citing the need for ongoing reflective support even after several months of practice. This suggests that programmes like ProDeveloP may need to be extended or offered more broadly, including more experienced RNs, to further support long‐term professional development. Reflection on clinical experiences and the sharing of knowledge are recognised as essential for deepening the understanding of the nursing profession and enhancing the ability to navigate complex work environments (Ekebergh et al. 2018; Widarsson et al. 2020). Onboarding strategies that incorporate structured, supportive introductions have been shown to increase role clarity and task mastery, ultimately reducing turnover rates (Gustavsson et al. 2020). Introduction programmes are also economically advantageous, as they significantly reduce the costs associated with turnover, such as expenses related to orientation, training, and ongoing support (Rush et al. 2019).

In addition to using the reflection opportunities provided in the introduction programme, ProDeveloP, the NGNs also engaged in reflection with experienced colleagues in the workplace to enhance task performance and manage their daily responsibilities. Support through spontaneous reflection with experienced RNs during shifts was vital for handling professional tasks and advancing their professional development. Novice nurses require guidance from experienced nurses to develop the core competencies and skills necessary for managing nursing care (Benner 2004). During the initial months of transitioning into their professional roles, NGNs often face challenges in meeting the responsibilities of their profession, particularly due to their limited experience and insufficient access to expertise and clinical support from other nurses (Duchscher 2008). In this study, the NGNs found that reflection with experienced RNs was crucial for maintaining patient safety, given the complexities of caring for patients with diverse needs. Nurse supervisors must support NGNs in assessing these complexities to ensure patient safety (Bindon 2017; Willman et al. 2021). Moreover, the engagement of RNs and their willingness to support NGNs in reflective practices were evident, particularly when the NGNs had an assigned RN as a clinical supervisor during their introduction phase. However, after this introduction phase, finding time for reflection in the fast‐paced environment became increasingly difficult. In instances where an experienced RN was unavailable, temporary RNs with more experience could offer reflective support to help manage the workload. The presence of experienced RNs in the workplace is essential for supporting NGNs during their transition (See et al. 2023; Widarsson et al. 2020). NGNs require professional support from experienced RNs, who can act as role models and facilitate their integration into the profession (Benner 2004).

The NGNs also expressed a need for opportunities to share experiences and knowledge, as well as for more dedicated one‐on‐one reflection with experienced RNs, including individual feedback on their progress–an opportunity that is seldom provided in the workplace. Those who had an assigned mentor with experience as an RN were better positioned to engage in more in‐depth reflection, receive emotional support, and benefit from individual professional development. Transitional mentoring plays a crucial role in supporting NGNs during this period (Jangland et al. 2021). Developing introduction programmes that better support NGNs in navigating their transition and addressing their need for reflection is essential, particularly in light of the current nursing shortage.

Besides reflection with more experienced RNs, this study found that the NGNs were socially included in an open, collaborative climate, fostering opportunities for spontaneous reflection with experienced colleagues. However, despite this inclusion, the environment was not experienced as trustworthy or conducive to structured, in‐depth reflection that could support sensitive, patient‐focused thoughts. The lack of trust and transparency led to superficial workplace reflections that did not meet the NGNs' needs. Previous studies have highlighted that NGNs face insufficient support in meeting expectations of holistic care (Kaldal et al. 2023; Widarsson et al. 2020). Our findings further reveal the problem NGNs experience in finding some opportunities for self‐reflection and peer reflection, and that they encounter barriers to meaningful development, often due to an unsupportive workplace structure. Research has constantly demonstrated that the lack of resources and structure impedes NGNs' professional growth (Hakvoort et al. 2022; Rudman et al. 2024).

It is critical that organisations allow NGNs to develop at their own pace, as factors such as reduced workload, stable staffing, and a supportive culture have been shown to facilitate their professional growth (Berglund et al. 2022). This study also highlighted a gap in managerial engagement, revealing a lack of formal structures for sharing experiences and knowledge, as well as for evaluation processes and follow‐up plans to support NGNs during their transition. Greater responsibility is needed from organisational leadership to ensure that NGNs are provided with the necessary conditions for growth, including personal goal recognition and adequate reflection opportunities. Personal goals and individual needs must be recognised, even though NGNs are often seen to lack support from managers (Hakvoort et al. 2022). Without such support, NGNs risk leaving not only the workplace but the nursing profession entirely, exacerbating the ongoing global nursing shortage (Kaldal et al. 2023; WHO 2022).

The findings from this study indicated that the ProDeveloP programme supported NGNs' professional development at an organisational level by providing structured, regular reflection sessions with opportunities to share experiences and knowledge. However, it lacked collaboration with local workplaces, which may explain the variability and scarcity of reflection opportunities in the workplace. The availability of workplace reflection often depended on the local organisation. Despite colleagues' willingness and engagement, challenges within the work environment frequently hindered these opportunities. The NGNs highlighted that reflection on experiences and knowledge directly related to their daily work and patient care was crucial for their professional development within specific contexts. The research underscores the importance of both workplace supervision and organised introduction programmes for effective clinical practice (Berglund et al. 2022).

To enhance professional development, learning needs to be integrated across both clinical practice and educational settings (Page et al. 2020). Reflection as a support for lifelong learning and professional growth should be incorporated into both introduction programmes and workplace settings. This suggests that shared responsibility between the workplace and organisational programmes is essential to effectively support NGNs during their transition. Integrating peer reflection and self‐reflection as core elements of learning would significantly enhance NGNs' professional development. The main positive outcome of this study is that engagement was experienced, and efforts were made to support NGNs during their transition. However, these efforts lacked the necessary prerequisites and structure in the workplace to be fully effective in supporting NGNs' professional development.

### 5.1 Strengths and Limitations

This study has several strengths. The use of focus groups to gather qualitative data allowed the NGNs to engage in collective discussions, providing rich insights into both their shared and individual experiences during the transition period. This approach, as opposed to individual interviews, facilitated dynamic exchanges, encouraging the participants to explore a broader range of perspectives (Morgan 1997). The face‐to‐face format was particularly valuable, as it enabled discussion. Additionally, holding the focus group interviews close to the early stages of the transition period minimised the risk of recall bias, ensuring more accurate reflections. The variation in the participants' age and gender was considered reasonable for the nursing profession.

Adhering to the COREQ checklist (Tong et al. 2007) was beneficial for ensuring transparency and thoroughness in reporting the qualitative focus group methodology. However, the study also has some limitations. While researchers attempted to schedule interviews at convenient times for the volunteers, not all the NGNs were able to participate due to work commitments, despite their managers' approval and their availability on their days off. The researchers' prior knowledge of the subject could have influenced the findings. Nevertheless, ethical considerations and researcher reflexivity throughout the process were strengths of the study. All phases of the analysis were thoroughly discussed with the research team, and the assistant moderator (the last author) verified the accuracy of the findings.

### 5.2 Recommendations for Further Research

These findings related to NGNs' transition support through reflection can be transferred and applied across various hospital settings globally. However, further studies in different contexts are necessary to determine how transferable these findings are to other environments. Future research should focus on understanding how reflection in introduction programmes such as group dialogues, in conjunction with workplace support, facilitates NGNs' professional development in diverse healthcare settings. The opportunity for experienced RNs to serve as facilitators in dialogue groups may contribute to the retention of experienced RNs, though more studies are needed in this area. Further research is also needed to explore the role of managers in supporting NGNs' professional development. Additionally, research should explore how such support influences NGNs' ability to manage work demands and recover from job‐related stress. Supporting NGNs to remain in their profession and workplace could lead to a more stable overall level of professional competence benefiting both the organisation and enhancing the quality of care and patient safety.

### 5.3 Implications for Policy and Practice

The findings of this study can inform decision‐making priorities for managers and policymakers regarding the role of reflection as a tool for supporting professional development. These findings can contribute to creating a sustainable model of professional growth, where NGNs are enabled to develop into experts in their field, ultimately enhancing both patient safety and the quality of care. By addressing the need for lifelong learning and professional development, the research highlights the importance of integrating reflection into the support structure for both NGNs and experienced nurses. Providing various opportunities for reflection may encourage nurses to remain in their profession and workplace. Retaining RNs strengthens overall competence within healthcare, fostering a safer and more stable environment for both staff and patients.

## 6 Conclusions

The combination of self‐reflection and structured reflection with others is crucial for supporting NGNs' professional development. Reflection, both within organised introduction programmes and in the workplace, is essential. Peer reflection in dialogue groups and through learning activities with health instructors provided the NGNs in this study with a safe space to reflect in an environment away from direct patient care, which proved valuable for their professional growth. A trusting environment was seen as vital for fostering deeper, more personal reflection. However, the NGNs also needed opportunities to reflect with experienced colleagues in the workplace when closely involved in patient care. These opportunities were often inconsistent due to a lack of workplace prerequisites. Managers must create the conditions necessary for regular and meaningful reflection. To fully support NGNs' development, reflection with both peers and experienced colleagues is critical as it enhances job satisfaction, professional growth, and retention within the profession. A cultural shift may be required to make reflection a routine part of healthcare practice, benefiting not only NGNs but all healthcare personnel.

## Author Contributions

Conceptualisation and methodology (M.L., J.H., A.L., M.L.S.K., M.A. and M.W.). Investigation (M.L. and M.W.). Formal analysis (M.L. and M.W.). Writing – original draft (M.L.). Writing – review and editing (M.L., J.H., A.L., M.L.S.K., M.A. and M.W.). Funding acquisition (M.A.).

## Conflicts of Interest

The authors declare no conflicts of interest.

## Supporting information


Data S1.

## Data Availability

The authors have nothing to report.
